# Supplementary material for: Wide range continuously tunable and fast thermal switching based on compressible graphene composite foams
Source: Nat Commun. 2021 Aug 13;12:4915. doi: 10.1038/s41467-021-25083-8 (PMC8363619; doi:10.1038/s41467-021-25083-8)
Supplement: Supplementary file 1 — Supplementary Information [file 41467_2021_25083_MOESM1_ESM.pdf]

Supplementary Information

Wide Range, Continuously Tunable, and Fast Thermal Switching Based on Compressible Graphene Composite Foams

Du *et al*.

Supplementary Notes

Supplementary Note 1: Spring model.

To understand the change in thermal conductivity of compressed graphene foam in MD simulations, we modeled the thermal behavior under compression of the 3D porous foam as that of a 1D spring. Shown in Supplementary Fig. 1, a spring at uncompressed state with a length x_u and a cross-section area A_s is placed between the hot and cold plates. Assume the material of the spring wire has the thermal conductivity k_w, the length L_w, and the cross-section area A_w, the effective thermal conductance at uncompressed state G_u can be expressed as:

$$G_u = \frac{k_u A_s}{x_u} = \frac{k_w A_w}{L_w}. \quad (1)$$

Similarly, when the spring is compressed to a length x_c, the effective thermal conductance is:

$$G_c = \frac{k_c A_s}{x_c} = \frac{k_w A_w}{L_w}. \quad (2)$$

The thermal conductance stays constant as the spring is compressed, since the length of the pathway that heat flows through in both cases is the length of the spring wire, assuming contacts between adjacent spring coils are not established during compression. Equating the Supplementary Equation (1) and (2) leads to the prediction of decreasing thermal conductivity with decreasing spring length.

Supplementary Note 2: Transient temperature response in 10 cycles.

We measured the evolution of temperature difference across the sample during 10 cycle on the environmental chamber setup between uncompressed state and fully compressed state at 0.086 mm. The time required for the temperature to rise/drop by 10 °C are 3.87±0.63 minutes and 1.21±0.25 minutes, respectively. Although experimental error cannot be avoided in the compression process, it can be demonstrated that the cycling performance of graphene foam is stable as evident in Supplementary Fig. 2.

Supplementary Note 3: Stress-strain relation of compressible graphene/PDMS foam.

The stress-strain relation of the foam sample has been measured using Instron mechanical tester (E-1000 series). We recently purchased a new batch of samples from the same vendor and used it for stress-strain testing. Some characteristics of the new sample differ slightly from that of the old

one in terms of thickness and maximum compressive strain. Potentially, the fabrication process from the vendor has certain variation. However, as the composition of the foam remains the same, we believe the mechanical test results of the new foam can represent the typical mechanical behaviors of the previously used one. For mechanical testing, the sample is compressed uniaxially from 1.467 mm to 0.734 mm (~50% compressive strain). Load is applied and released at constant velocity of 0.01 mm s^{-1} and some hysteresis observed on compression versus release. In Supplementary Fig. 3, the stress in the release path is greater than 0 when reaching 0 strain, meaning the sample fully recovers to its original thickness from 50% compressive strain. The two paths differ slightly with reduced stress at the same strain level in release path. Similar mechanical behavior was reported in literature including for graphene grown with a CVD method on nickel foam then coated with PDMS[1], a 3D graphene network grown on porous ceramic SiO_2 substrate[2], and a 3D graphene formed in pyrrole-containing graphene oxide suspension[3].

Supplementary Figures

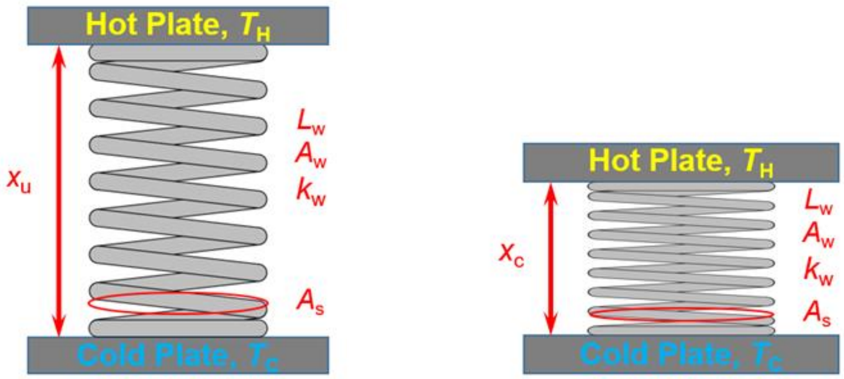

Supplementary Fig. 1. Schematic of the spring model. The 3D foam is modeled as a 1D compressible spring between the hot and cold reservoirs. From uncompressed to compressed states, the cross-section area of the spring, the length of the spring wire, and the cross-section area of the spring wire stay constant.

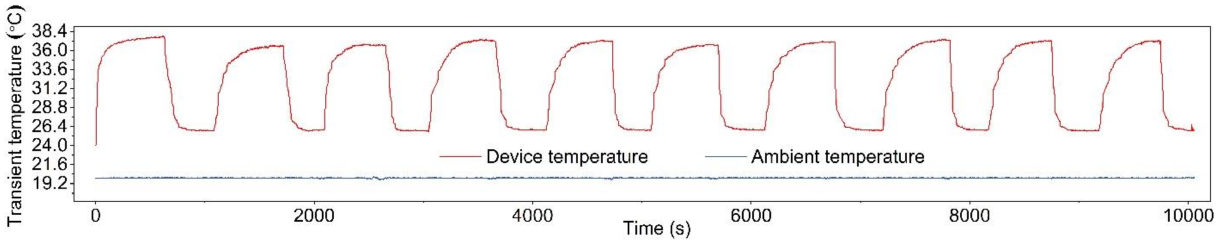

Supplementary Fig. 2. Transient temperature response in 10 cycles. The evolution of temperature difference across the sample in 10 cycles is measured on environmental chamber apparatus at an ambient temperature of 20 °C. Small variation is observed in temperature drop from uncompressed to fully compressed state among 10 cycles.

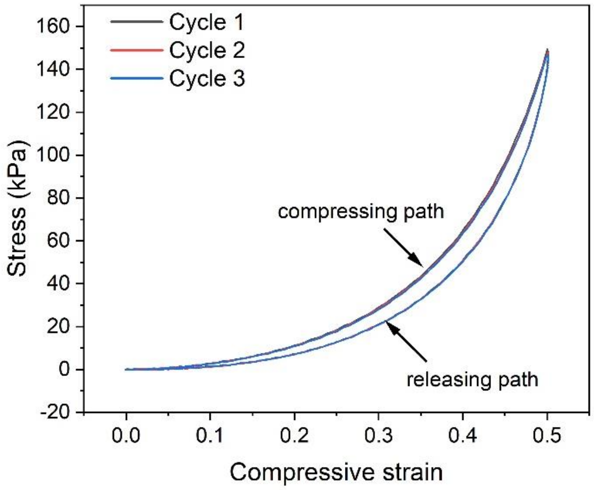

Supplementary Fig. 3. Stress-strain relation of compressible graphene/PDMS foam. The stress is measured with compressive strain for 3 compressing-releasing cycles. In both paths, the measured stress shows little deviation across 3 cycles.

Supplementary References

1. Chen, Z., Xu, C., Ma, C., Ren, W. & Cheng, H. M. Lightweight and flexible graphene foam composites for high-performance electromagnetic interference shielding. *Adv. Mater*. **25**, 1296-1300 (2013).

2. Huang, H., Bi, H., Zhou, M. Xu, F., Lin, T., Liu, F., Zhang, L., Zhang, H. & Huang, F. A three-dimensional elastic macroscopic graphene network for thermal management application. *J. Mater. Chem. A*. **2**, 18215 (2014).

3. Zhao, Y., Liu, J., Hu, Y., Cheng, H., Hu, C., Jiang, C., Jiang, L., Cao, A. & Qu, L. Highly compressible-tolerant supercapacitor based on polypyrrole-mediated graphene foam electrodes. *Adv. Mater*. **25**, 591-595 (2013).
