# Supplementary material for: Wide range continuously tunable and fast thermal switching based on compressible graphene composite foams
Source: Nat Commun. 2021 Aug 13;12:4915. doi: 10.1038/s41467-021-25083-8 (PMC8363619; doi:10.1038/s41467-021-25083-8)
Supplement: Supplementary file 5 — Description of additional supplementary files [file 41467_2021_25083_MOESM5_ESM.docx]

Description of additional supplementary information

Title: Supplementary Movie 1

Description: The compression process of graphene-PDMS foam in experiment

Title: Supplementary Movie 2

Description: The compression process of graphene foam in molecular dynamics simulations
